# Micro CT analysis of the subarticular bone structure in the area of the talar trochlea

**DOI:** 10.1007/s00276-012-1069-x

**Published:** 2013-01-10

**Authors:** Andrej Maria Nowakowski, Hans Deyhle, Silvan Zander, André Leumann, Magdalena Müller-Gerbl

**Affiliations:** 1 Orthopedic Department, University of Basel, Spitalstrasse 21, 4031 Basel, Switzerland; 2 Anatomical Institute, University of Basel, Pestalozzistrasse 20, 4056 Basel, Switzerland; 3 Biomaterials Science Center, University of Basel, Schanzenstrasse 46, 4031 Basel, Switzerland

**Keywords:** Loading history, Trabecular meshwork, Numerical parameter, Structural parameter, Micro CT

## Abstract

**Purpose:**

Certain regions of the talar trochlea are recognized as exhibiting varying cartilage thickness and degrees of subchondral bone mineralization. These changes have been attributed to the long-term loading history. For the current study, we accepted the hypothesis that stress-induced alterations of the joint surface include not only varying degrees of subchondral lamellar mineralization, but also structural changes of the subarticular cancellous bone.

**Methods:**

In order to examine the structure of the subarticular cancellous bone, ten formalin-fixed talar trochleae were analyzed using micro CT. Sixteen measurement zones were defined and then evaluated in five layers each of 1-mm thickness, enabling assessment of the cancellous architecture extending 5 mm below the trochlear surface using numerical and structural parameters.

**Results:**

As with mineralization patterns in the subchondral lamella, large variation was observed regarding bone volume, trabecular quantity, thickness, and spacing, as well as for structure model index and degree of anisotropy, depending on localization. In addition, like previous reports examining mineralization of the subchondral lamella, two distinct groups could be identified as “bicentric” or “monocentric”.

**Conclusions:**

These results show that structural tissue adaptation probably due to loading history is also evident within the subarticular cancellous bone.

## Introduction

The articular surfaces of the upper ankle are stressed primarily by axial compression through a vertical load vector [10]. However, bending load is also exerted by tension from the collateral ligaments [18].

Close [3] and Fick [7] reported increasing intermalleolar distance on dorsiflexion of the foot. This also exposes the talus to flexural stress. Similarly, Calhoun et al. [2] found that under stress in dorsiflexion, the medial and lateral contact areas of the malleolar articular surfaces are enlarged.

Because of physiological incongruence, only certain parts of the opposing talocrural articulating surfaces are available to absorb load [10]. In previous studies of various joints, Muller-Gerbl [9, 11] reported that the extent of mineralization and its distribution in the subchondral tissues are not homogeneous. Thus, areas of increased mineralization are evident, and these zones correspond to areas absorbing stronger mechanical forces. Using distribution patterns of subchondral mineralization (densitograms), the burden on the joint surface can be measured over extended periods of time as a “loading history” [10]. Particular patterns of mineralization seem to correlate with the talar profile. At the deeper trochlear groove, usually a bicentric pattern occurs, while the flattened portion is associated with a monocentric pattern [10].

Pal and Routal [14] and Ebraheim et al. [4] examined the trabecular architecture of the talus radiographically with cuts in three levels, and reported a varied distribution of cancellous bone trabeculae with different course distribution in particular regions of the talus. Athavale et al. [1] recorded similar observations after evaluating three-dimensional (3D) serial cuts.

With the advent of quantitative determination of the structural parameters of trabecular bone using micro CT techniques [6, 13, 15], a high-resolution method for 3D visualization and analysis of the micro-architecture of human bone is now available. The current work uses these micro CT techniques to investigate whether load-induced adaptations, comparable to the varying cartilage thickness and degrees of mineralization seen in the subchondral lamella, occur histologically within the subarticular cancellous bone.

## Materials and methods

Five right and five left talar specimens were obtained from the anatomic dissection course for medical students at the University of Basel (2006–2008). The human cadavers were immediately fixed with intra-arterial formalin application on arrival at the Anatomical Institute. According to their legal testaments, donors provided their bodies to the Anatomical Institute after death for the purposes of science and research. Among the donors were four men and six women aged 72–91 years (average age 85.4 years).

**Fig. 1:**
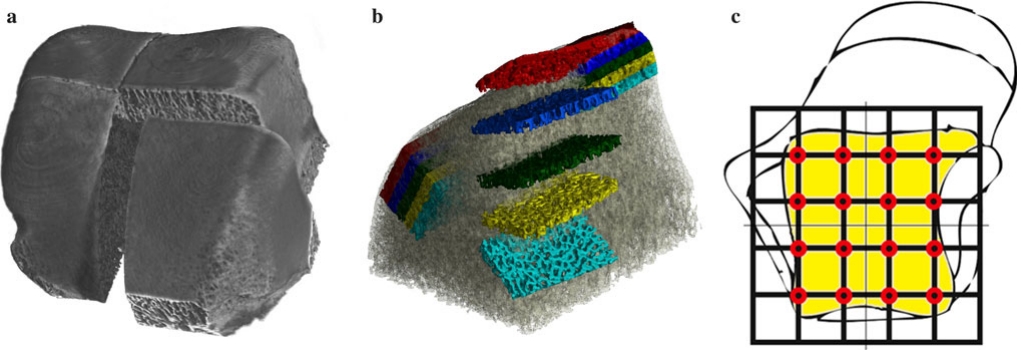
Definition of measured zones: **a** 3D reconstruction of the Micro CT data of an initially quartered talar trochlea. **b** Representation of the 5 × 1 mm thick layers as five separate volume elements. Each talar quarter from Fig. 1a was divided into four areas, one of them shown here as an expanded view for better visualization. **c** The 4 zones from **b** on the 4 talar quarters from **a** yields 16 zones in 5 layers, thus a total of 80 separate volume elements

Initially, the tali were removed from the cadavers, and the talar trochlea were then prepared as follows: After removal of all ligaments, the anterior talar neck and talar head were detached. Posteriorly, the talus was divided horizontally, just superior to the posterior tubercle. Due to size constraints for the micro CT assessment, the trochleae were further divided into four segments (Fig. 1a) .To minimize material loss from bone cuts, a diamond-blade saw was used (Exact Band System 310, Norderstedt, Germany, saw blade 0.3 mm/D151).

The trochlear quarters were scanned separately with Micro CT (Skyscan 1174™, Kontich, Belgium). Within the cone beam device with micro-focus tube, the acceleration voltage was 50 kV at a beam current of 800 μA (in total 40 scans). In addition, a 0.5 mm thick aluminum filter was used to minimize beam hardening. Each scan included 900 projections from 360°. The pixel size of the resulting images was 32 μm isotropic.

The first step for volumetric reconstruction of the raw micro CT data used NRecon™ software (Skyscan, Nrecon reconstruction, Kontich, Belgium), which is based on a modified Feldkamp algorithm. A specially programmed algorithm using MATLAB^®^ software (version 5.3 from The MathWorks Inc., USA) was then used for each investigated data set to define columnar measuring segments for analysis of the structural parameters. The base of each column extended 7 mm sagittally, while the dimensions in the anterior plane depended on the specimen size. Height measured 5 mm, divided into five volume elements each of 1 mm (Fig. 1b).

The measured columns are perpendicular to the transverse axis, and tilt according to the trochlear radius in the sagittal plane, so that they can be considered approximately perpendicular to the surface of the talar trochlea.

The five 1-mm sections of each column correspond to the volume elements: layer 1 (0–1 mm), layer 2 (1–2 mm), layer 3 (2–3 mm), layer 4 (3–4 mm), and layer 5 (4–5 mm). Four columns per sample were examined, yielding 16 measurement columns per talar trochlea (Fig. 1c). Within each of these 16 zones were the five volume elements, thus yielding a total 80 volumes analyzed per talus.

**Fig. 2:**
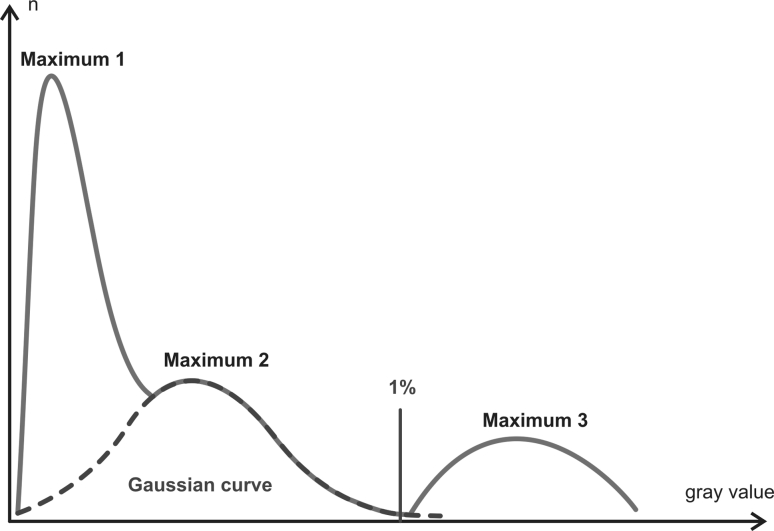
*Gray* value histogram of a quartered talar specimen. *Maximum 1* air, *Maximum 2* container, *Maximum 3* mineralized bone. Setting of the threshold where the interpolated *Gaussian curve* of the container drops from 1 % of their maximum

For further analysis, the CT Analyzer (version 1.7.0.5, Skyscan, Kontich, Belgium) program was used. For calculation of the morphometric parameters, initially bone and the surrounding tissues were binarized. For this, a threshold value for each talus was determined using a histogram of the respective data (Fig. 2).

The gray scale values of the CT analysis showed 3 maxima on the histogram, each denoting an area of absorption. Maximum 1 corresponds to the medium (air), and maximum 3 to the highly absorbent mineralized bone. The intervening, more weakly pronounced maximum 2 reflected the plastic tank used to hold the sample. The margins of the Gaussian curves were overlapping. The maximum corresponding to the container material was completed with a Gaussian curve. The threshold was defined at the point on the curve reaching 1 % of the maximum value. All voxels with more intense gray values were attributed to bone.

**Fig. 3:**
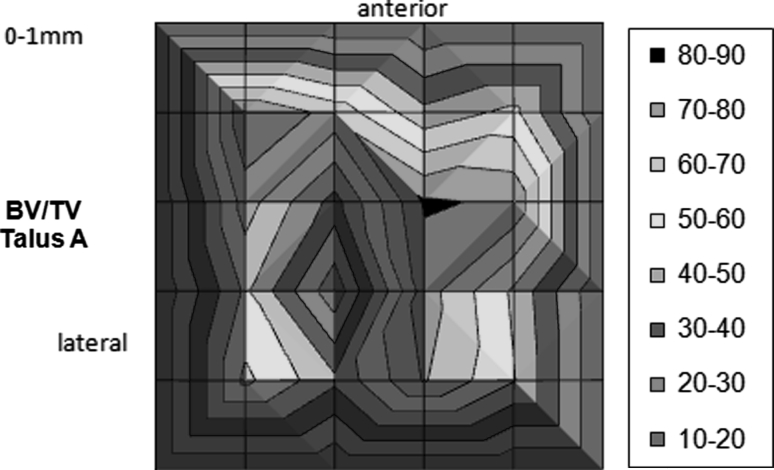
Graphical representation of the BV/TV parameters for all 16 zones of a single layer (0–1 mm) of talus A

This protocol was followed, and six parameters for cancellous bone were calculated in each volume element: bone volume/tissue volume (BV/TV), structure model index (SMI), trabecular thickness (Tb.Th), trabecular number (Tb.N), trabecular separation (Tb.Sp), and degree of anisotropy (DA). Next, the distribution of the measured values was mapped out based on the locations of maximum and minimum values. As an example, Fig. 3 shows a corresponding representation of the parameter BV/TV for all 16 measurement areas of one layer (0–1 mm) of talus A.

**Fig. 4:**
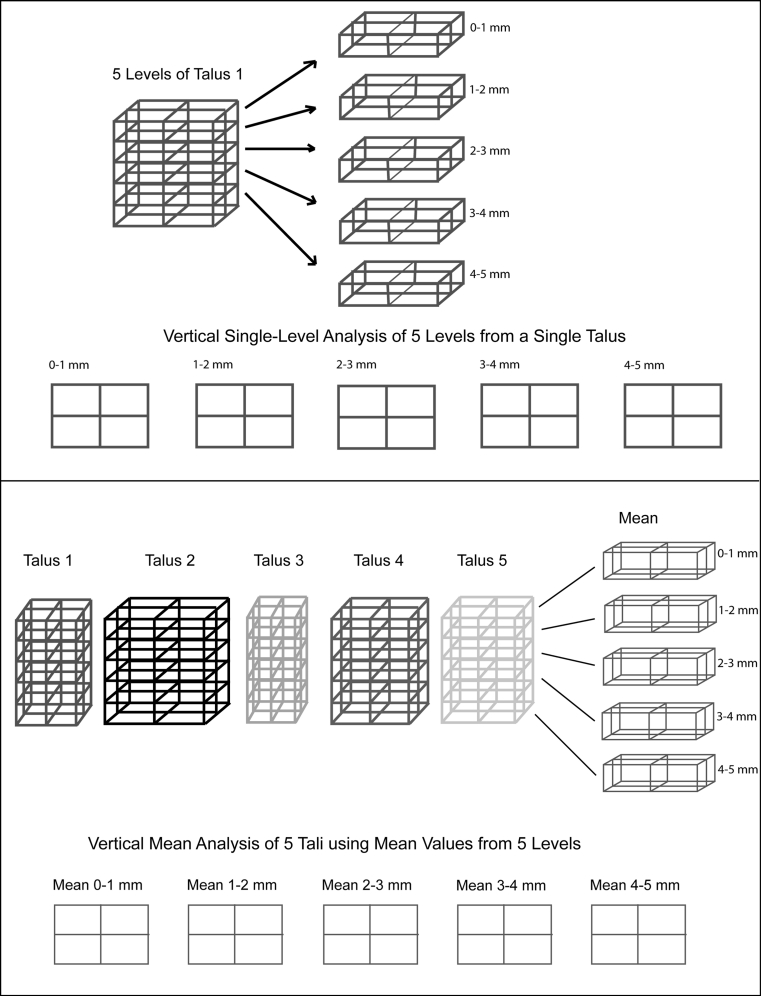
Vertical single layer and mean value analysis represented schematically. The *different colors* and *sizes* represent the *individual* and *varied talar shapes*

**Fig. 5:**
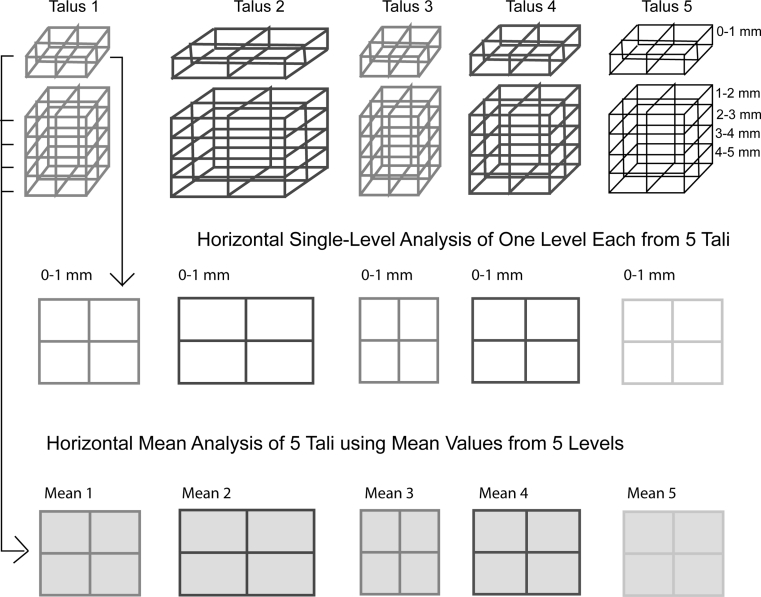
Horizontal single layer analysis and mean value analysis represented schematically. The *different colors* and *sizes* represent the *individual* and *varied talar shapes*

To analyze the multitude of images (10 tali, 5 levels, 6 parameters = 300 images), geographic representations of average values were created, consisting of the average values from “vertical” levels of the talus (Fig. 4), as well as the average values of a prototypical distribution group on a single plane to reflect the “horizontal” map (Fig. 5).

**Fig. 6:**
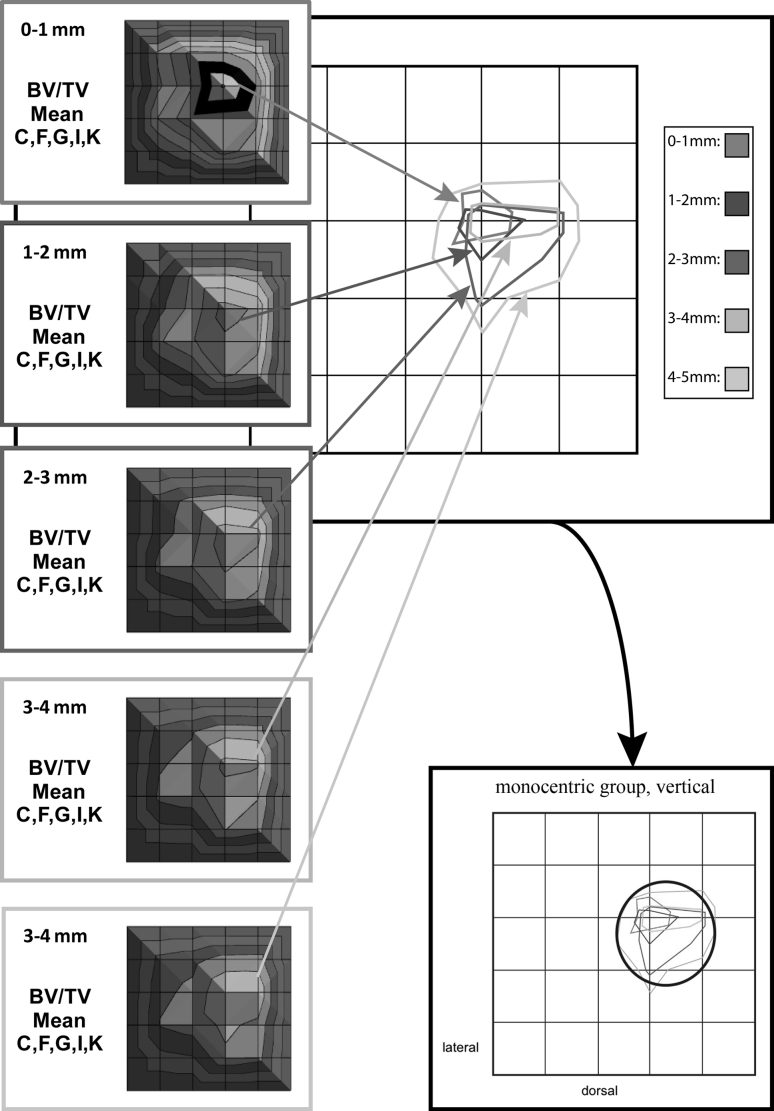
Summary of maximum values from the mean value graphs to produce a summation scheme

Finally, the maxima of the average values were merged for a “synoptic” look. For this purpose, the geographic representations were subsequently schematized using software (Illustrator CS2, Adobe^®^, US), so that the fields of the respective maximum values were projected over each other (Fig. 6).

## Results

For all ten talar specimens, the complete trochlear surface was standardized to examine the 16 localized zones. For each of these zones, five discrete depths (in 1 mm increments descending from the surface) were evaluated. Thus, beneath each superficial projection zone, vertical columns of 5 mm depth were analyzed. This yielded measurement values from 800 volume elements. Graphic depiction of the micro CT measurements yielded 50 images per parameter.

### Numerical parameters

On horizontal single layer analysis, the numerical parameters indicated two distinct patterns of talar architecture: monocentric and bicentric. Each pattern was evident in five of the ten tali:

- Bicentric distribution pattern: tali A, B, D, E and H, and
- Monocentric distribution pattern: tali C, F, G, I and K.

The results for the numerical parameters are shown separately, according to distribution pattern.

#### Bone volume/tissue volume (BV/TV)

**Fig. 7:**
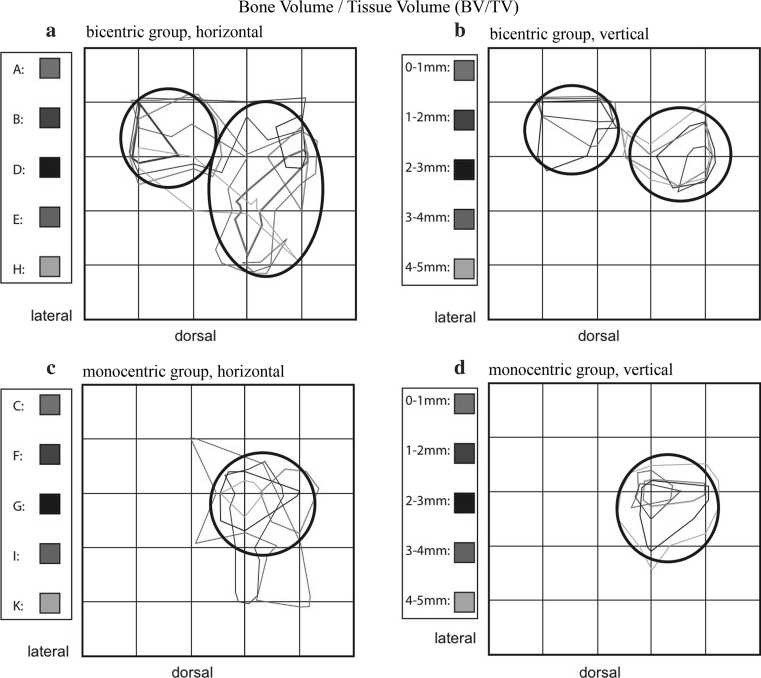
Summation schemes for the numerical parameters of bone volume/tissue volume (BV/TV). Bicentric (**a**, **b**) and monocentric (**c**, **d**) distribution patterns can be distinguished from one another

On horizontal analysis of the bicentric group, all images showed a heterogeneous distribution of mineralized bone. Also noted were regions of greater bone volume fraction. Looking more specifically at these consolidated zones, maxima are concentrated in a smaller area ventrolaterally, and within a larger region in the medial half of the trochlea (Fig. 7a).

In the vertical analysis schema, the zones of compression are also focused within two fields (Fig. 7b). The ventrolateral zone is in almost the identical position as that described for the horizontal analysis, while the ventromedial center is more limited. Vertical comparison of all five layers shows decreasing maximum values with increasing field size in deeper layers, from red 0–1 mm up to gray 4–5 mm.

In the monocentric group, horizontal analysis of all five layers identifies a zone of increased bone volume fraction centromedially, at the transition from the central to the ventral third of the trochlear surface, in all samples (Fig. 7c).

Summation of vertical results yields approximately the same location (Fig. 7d). In addition, vertical analysis yields maximum values concentrated in smaller fields, which clearly overlap one another. Also in this group, fields are larger in the deeper layers, although the absolute magnitude decreases. In addition, horizontal analysis of this group shows more polymorphic field definition and a less marked narrowing of the described central zone.

#### Trabecular number (Tb.N)

**Fig. 8:**
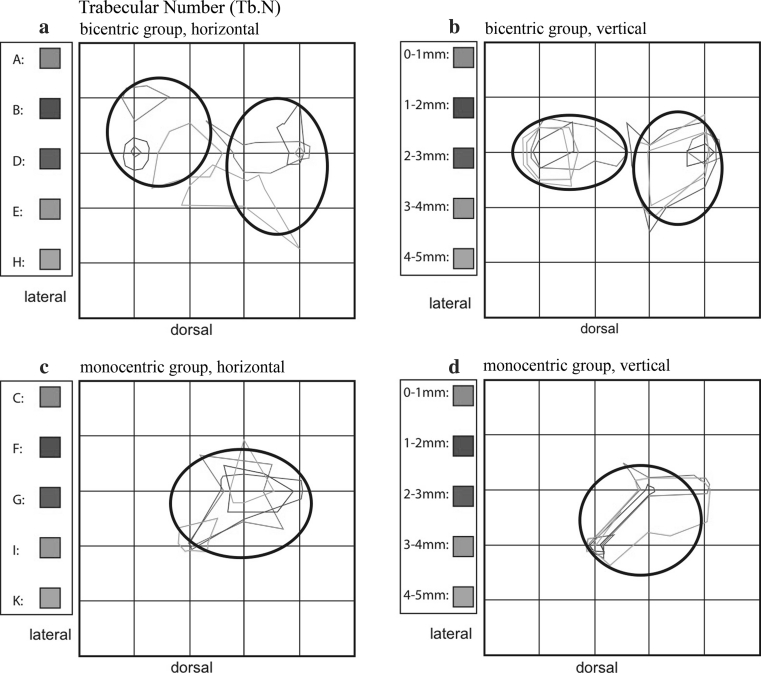
Summation schemes for the numerical parameter trabecular number (Tb.N). Like BV/TV, the bicentric distribution pattern (**a**, **b**) is distinct from the monocentric (**c**, **d**)

For this parameter also, a bicentric distribution pattern of maximum values is evident in specimens A, B, D, E, and H. The locations of the maxima are determined in the same manner as those of BV/TV (Fig. 8a). With increasing depth of the analyzed tissue layers, the magnitude of the measured maximal values decreases (Fig. 8b).

A monocentric distribution is evident for tali C, F, G, I, and K in a similar position to the maxima of the BV/TV parameter (Fig. 8c). Again, a relative decrease of the maximum values was identified with increasing depth (Fig. 8d).

#### Trabecular thickness (Tb.Th)

**Fig. 9:**
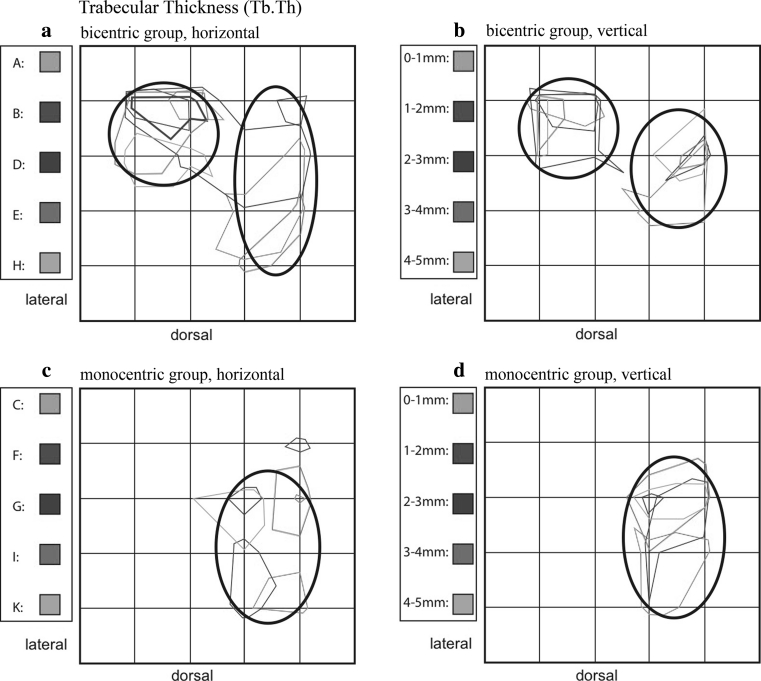
Summation schemes for the numerical parameter Trabecular thickness (Tb.Th). Like BV/TV and Tb.N, the bicentric distribution pattern (**a**, **b**) is distinct from the monocentric (**c**, **d**)

Evaluation of this parameter also identified a bicentric distribution pattern for tali A, B, D, E, and H, with maxima ventrolaterally and medially (Fig. 9a), in large part corresponding to the distribution for BV/TV. Again, a relative decrease of the maximum values was identified with increasing bony depth (Fig. 9b).

In tali C, F, G, I, and K, a monocentric distribution was evident with zones also corresponding to those of BV/TV (Fig. 9c). Horizontal analysis also showed more polymorphic field definition than vertical analysis, with scattered, smaller islands of comparable maximum values. As described for BV/TV and Tb.N, areas of the maximum values can be more clearly localized on the vertical analysis (Fig. 9d).

#### Trabecular separation (Tb.Sp)

**Fig. 10:**
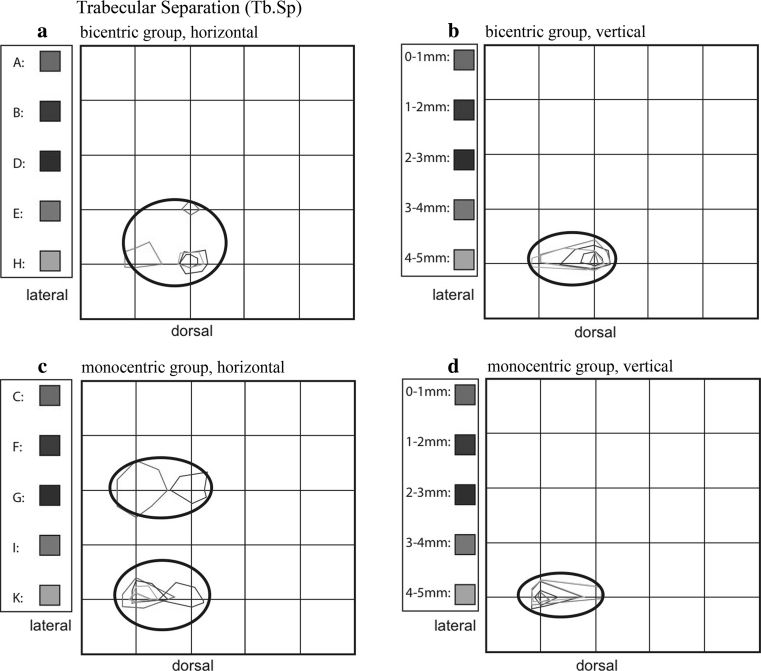
Summation schemes for the numerical parameter trabecular separation (Tb.Sp). In contrast to the preceding numerical parameters (BV/TV, Tb.N, TB.Th), the distribution patterns here are dorsolaterally located (**a**–**d**), and the descriptive mapping of bicentric versus monocentric appears inversed

In the bicentric group, the maximum values are concentrated dorsolaterally, in contrast to the preceding parameters (Fig. 10a, b).

In the five tali with a monocentric distribution pattern, the subarticular cancellous trabeculae are also increasingly separated dorsolaterally, as well as within a centrolateral area (Fig. 10c). With increasing layer depth, a decrease of the maximum values was seen only in the monocentric pattern (Fig. 10d).

### Structural parameters

When comparing all ten tali in terms of structural parameters, the distribution appears more homogeneous than the previously described numerical parameters.

#### Structure model index (SMI)

For the diagrams, we will maintain the separate consideration of the bicentric and monocentric groups.

**Fig. 11:**
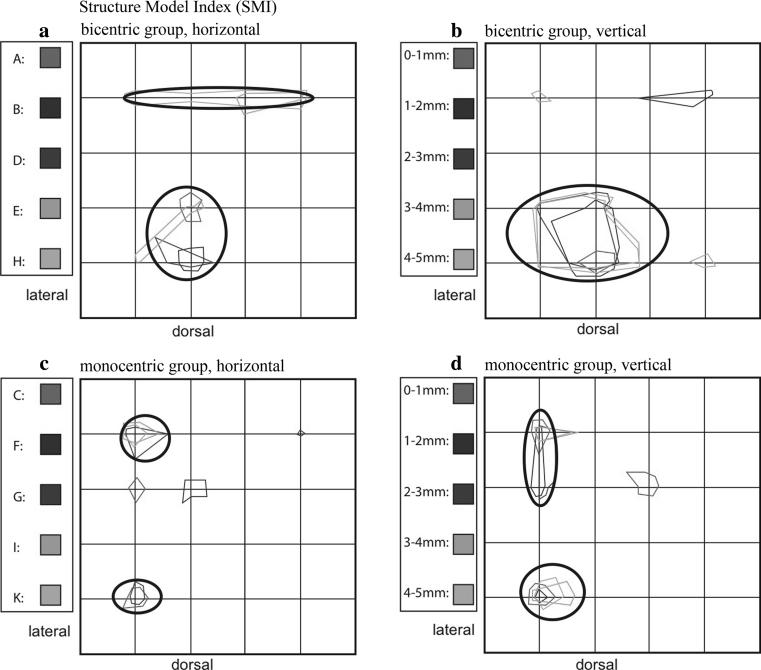
Summation schemes for the structural parameter structure model index (SMI). Compared to the numerical parameters, the SMI values are relatively homogeneously distributed within the trochlea. Higher values for SMI were measured, especially in the lateral half of the talus

For tali A, B, and D of the bicentric group, the SMI values increased dorsolaterally (Fig. 11a, b). E and H show additional maxima ventrally.

For the monocentric tali C, F, I, and K, the maxima are more common in the lateral half of the talus. The ratio of plate and rod elements in the tali of this group appears relatively balanced, particularly with increasing depth, but with a tendency for rod-rich areas dorsolaterally and ventrolaterally (Fig. 11c, d).

#### Degree of anisotropy (DA)

All ten tali show heterogeneity with no conspicuous tendencies toward a structural concentration. Neither horizontal nor vertical analysis indicates regularities; the maxima distribution appears random. For most of the tali, a diffuse, multi-centric distribution is evident. On vertical comparison by depth, very little congruence is observed. Thus, it is difficult to make any conclusions based on this parameter.

## Discussion

For the in vitro evaluation of bony structures, macroscopically descriptive or bone densitometry is available. For the microscopic examination, for example, histomorphometric techniques can be used. The evaluation of thin sections is a decades-long established method to quantify structural parameters such as trabecular thickness and spacing.

The disadvantage of these procedures, in addition to the elaborate sample preparation required, is that for interpretation of the 2D sections, a model for extrapolation to the third dimension is required, and is not always successful [5].

Micro computer tomography was recently developed as an alternative to histomorphometry. In the original technique, technical challenges as well as limitation to relatively small sample volumes make it difficult to achieve non-destructive evaluation of bony architecture. In addition, with micro CT, the considerable efforts required for preparation, staining, and/or radiological evaluation of serial thin sections can be dispensed with.

According to Engelke et al. [5], with smaller trabecular volumes, the values of histomorphometric structural parameters can vary between 10 and 20 percent. With micro CT, the assessment of histomorphometric and other structures is dependent on imaging process resolution and the exponentially increasing amounts of data occurring at higher resolutions.

When the spatial resolution of micro CT data sets is artificially depreciated due to averaging over multiple voxels, the absolute number of errors increases. However, the linear relations between measurement error and resolution are known within certain limits, and such errors can be reduced by implementing the corresponding correction factor [12].

In addition, comparative measurements between micro CT and quantitative CT have shown good correlation for certain parameters (BV/TV, Tb.N, Tb.Th and Tb.Sp) [8]. Thus, these parameters seem well suited for assessments of the histologic bony structure in the μm range up to an isotropic resolution of 170 μm.

In the current study, the micro CT-generated data was averaged and then depicted visually to demonstrate the distribution of minimal to maximal measured values across the contour of the evaluated talar trochleae. The synoptic view of the corresponding levels of several distinct tali allowed comparison and contrast of samples with varied characteristics. In this way, the characteristics of the bicentric and monocentric distribution pattern groups could be filtered and presented.

To allow comparison of the diversity represented in the initial, multi-colored depictions, the zones of the maximal values were transferred onto a schematic chart. Overlapping projection of these schematically-represented fields is much clearer and allows better visualization of particular characteristics (summation schemes).

Particularly for the numerical parameters BV/TV, Tb.N and Tb.Th, the results of this work yield two distinct patterns expressing the heterogeneous distribution of maximum values. These parameters are generally associated with cancellous areas of greater density. Of the ten tali studied, five showed the bicentric and five a monocentric distribution pattern.

In contrast, the numerical parameter Tb.Sp identified increased separation of the trabecular structures. This parameter is associated rather with less dense areas (loose cancellous bone). Consequently, such areas have been identified mainly in regions where the previously mentioned parameters indicate no bony compression. This applies equally for the superficial and deeper bony layers.

The structural parameters of SMI, however, indicate the distribution of trabecular plate and rod structures. In our analysis, areas of increased rod distribution were seen particularly in the lateral half of the talus. In the monocentric group, they were concentrated both ventrolaterally and dorsolaterally, and in the bicentric group preferentially dorsolaterally.

Only analysis of the second structural parameter, DA, yielded no evident distribution pattern. Zones with higher maximum values, indicating an increased unidirectional orientation of trabecular structures, were evident. However, in all ten of the specimens, these areas varied in size and were variously distributed throughout all five tissue layers.

The depicted results, and in particular the distribution patterns of the maximum values of the four numerical parameters, correlate with those of previous reports.

Pal and Routal [14] reported that cancellous trabecular structures in the human talus align along suspected lines of force. They identified vertical plate structures oriented primarily dorsoventrally. These structures were connected by an irregularly-aligned network of bony trabeculae, which appeared to react to changes in the character and direction of acting forces.

According to Athavale et al. [1], cancellous plates can be found predominantly in the dorsal two-thirds of the lateral half of the trochlea, extending vertically down to the calcaneus. However, the course of other trabecular structures begins in the ventral third of the trochlea and the central parts of the medial half, mainly in the ventral direction to the neck and head of talus, towards the navicular bone.

In a previous study, Muller-Gerbl [10] identified the thickest layers of cartilage on the talar trochlea along the medial edge of the dome, as well as in a central area on the lateral side. In addition, they presented the distribution of subchondral mineralization on the talus most commonly as a bicentric pattern with maximal values in the area of the ventromedial and ventrolateral trochlea. The monocentric distribution pattern, however, showed areas of maximal mineralization ventromedially or at the approximate center of the trochlea.

The results of the current study show that structural tissue adjustments due to loading history are probably also evident within the subarticular cancellous bone. Typical distribution patterns are present which correspond to the cartilage thickness or to the mineralization of the subchondral bony lamella.

### Limitations of study

The absolute extent of bony mineralization differed at the individual level. Various explanations for this are possible, e.g., pathologically-related changes affecting bone metabolism as a whole such as osteoporosis, hormonal disturbances, or immobilization, but also physiological differences such as physical activity, weight, gender, age, etc.

This means that the level of mineralization as a whole is affected by general factors (endocrine, metabolism-related or activity-dependent); however, the distribution of the density and structure within the joint surface is affected by local mechanical factors (especially long-term loading history).

In addition, the small quantity of only ten talar specimens and lack of detailed documentation regarding the donors’ lifestyles, etc., do not allow definitive interpretation of the inter-individual measurement results.

Whether the bicentric and monocentric distribution patterns identified here are the result of corresponding distributions within the subchondral bone layers should be determined by further studies comparing mineralization distributions of the subchondral bone plate measured with computed tomography osteo absorbtiometry (CT-OAM). Such studies could also address additional questions, e.g., relating to relative axial malposition. Racial differences have also been observed in this area. Examining 300 Indian tali, Sharada et al. [17] identified a differing distribution of classified joint surface types compared to those of Egyptian and Spanish studies. In addition, a number of anatomic variants were reported, which according to Shahabpour et al. [16] could have implications for mobility and stability of the ankle.

## Conclusions

The synoptic view of the corresponding levels of several distinct tali allowed comparison and contrast of samples with varied characteristics. In this way, the characteristics of the bicentric and monocentric distribution pattern groups could be filtered and presented.

The regular, reproducible distribution patterns showing structural adjustment of the subarticular cancellous bone correlate with studies of long-term stress to the talar trochlea. Thus, the results of this study indicate that structural tissue adjustments probably due to loading history are also evident histologically within the subarticular cancellous bone.
